# Rapid kinetics of changes in oxygen consumption rate in thrombin-stimulated platelets measured by high-resolution respirometry

**DOI:** 10.1016/j.bbrc.2018.08.031

**Published:** 2018-09-18

**Authors:** Alice P. Sowton, Sarah L. Millington-Burgess, Andrew J. Murray, Matthew T. Harper

**Affiliations:** a Department of Pharmacology, University of Cambridge, UK; b Department of Physiology, Development and Neuroscience, University of Cambridge, UK

**Keywords:** Platelet activation, Oxygen consumption, Cyclo-oxygenase, Mitochondria, Respirometry

## Abstract

Platelet activation plays a key role in normal haemostasis and pathological thrombosis. Platelet activation is rapid; within minutes of stimulation, platelets generate bioactive phospholipids, secrete their granule contents, activate integrins and aggregate together to form a haemostatic plug. These events are dependent on ATP synthesis. Mitochondrial function in platelets from healthy volunteers and patients with a range of diseases indicate an important role for oxygen consumption in oxidative phosphorylation in normal and pathological function. Platelets also consume oxygen during oxidation reactions, such as cyclooxygenase-dependent thromboxane A_2_ synthesis. In this study, we used high-resolution respirometry to investigate rapid changes in oxygen consumption during platelet activation. We demonstrated a rapid, transient increase in oxygen consumption rate within minutes of platelet stimulation by the physiological activator, thrombin. This was partly inhibited by aspirin and by oligomycin. This shows that high resolution respirometry can provide information regarding rapid and dynamic changes in oxygen consumption during platelet activation.

## Highlights

• High resolution respirometry can be used to investigate the rapid kinetics of changes in platelet oxygen consumption rate.
• Thrombin triggers a rapid, transient increase in platelet oxygen consumption rate.
• Aspirin and oligomycin partially inhibit the increased oxygen consumption rate.

## 1 Introduction

Platelet activation is central to haemostasis and arterial thrombosis [1]. Platelets adhere at sites of vascular injury, generate and release bioactive phospholipids, release their granule contents through exocytosis, and activate their major integrin, α_IIb_β_3_. These events lead to recruitment of further platelets and platelet aggregation, forming a haemostatic plug or occlusive thrombus [1].

Platelet activation requires ATP, which is provided by increased mitochondrial oxidative phosphorylation (OXPHOS) and glycolysis [[2], [3], [4], [5]]. Pioneering experiments starting in the 1970s using Clark-type oxygen electrodes showed that platelet activation with a range of physiological stimuli triggers a rapid increase in O_2_ consumption, attributed to increased mitochondrial OXPHOS (inhibited by oligomycin) and oxidation of arachidonic acid by cyclooxygenase (COX; inhibited by aspirin) [6,7]. However, Clark-type electrodes show a number of problems, including uniform signal drift, changes in sensor response, and bubble formation on the electrode [8]. Importantly, the resolution achievable with Clark-type electrodes is relatively low compared to high-resolution approaches currently available.

More recent methodologies for studying O_2_ consumption, such as the Oxygraph-2k high-resolution respirometer (Oroboros Instruments) and the high-throughput Seahorse Extracellular Flux (XF) Analyzer (Seahorse Bioscience Inc.), have stimulated renewed interest in the regulation of O_2_ consumption in many primary cell types. Each system has its own advantages and disadvantages [compared in detail in Ref. [9]]. The Seahorse XF has been used to characterise platelet O_2_ consumption, mitochondrial function and glycolysis. This has usually been studied in unstimulated platelets, either from healthy donors [[9], [10], [11]], or from patients with conditions such as sickle cell disease [12], pulmonary hypertension [13], type 2 diabetes [14] or preeclampsia [15]. A small number of studies have reported changes in platelets following stimulation with thrombin, which increased O_2_ consumption rate by approximately 25% at the next measured time (approximately 8 min later) [3,16]. By this time, many of the rapid platelet functional responses have already occurred, including granule secretion and integrin activation [17]. Platelet O_2_ consumption and mitochondrial function have also been studied in the Oxygraph-2k. These studies have focused on respiration in unstimulated platelets from healthy donors [18], patients with sepsis [19] or Alzheimer's disease [20], and in response to drug treatment [21,22] or high-intensity training [23]. However, we are not aware of any studies that report the kinetics of rapid changes in O_2_ consumption using the Oxygraph-2k system in response to platelet stimulation with physiological activators such as thrombin.

In this study, we used the Oxygraph-2k to investigate changes in platelet O_2_ consumption following stimulation by thrombin. We describe a rapid, transient increase in O_2_ consumption rate that is partly dependent on COX activity and OXPHOS, but also depends on other pathways. This shows that high-resolution respirometry can provide information regarding rapid and dynamic changes in O_2_ consumption during platelet activation.

## 2 Methods

### 2.1 Materials

All reagents were purchased from Sigma unless otherwise specified. Amidated peptides, SFLLRN-NH_2_ and AYPGKF-NH_2_, were from Bachem (Weil am Rhein, Germany).

### 2.2 Washed platelet preparation

Blood was drawn by venepuncture into sodium citrate-containing vacutainers (3.8% v/v) from healthy, drug-free volunteers, who had given written, informed consent in accordance with the Declaration of Helsinki. Use of human blood from healthy volunteers was approved by the Human Biology Research Ethics Committee, University of Cambridge. Acid citrate dextrose (85 mM tri-sodium citrate, 71 mM citric acid, 111 mM d-glucose) was added (1:7 v/v) and platelet-rich plasma (PRP) separated by centrifugation (200 g, 10 min, room temp., no brake). Prostaglandin E_1_ (100 nM; Cambridge Bioscience) and apyrase (Grade VII; 0.02 U/ml) were added to PRP to prevent platelet activation during preparation. Platelets were pelleted from PRP by centrifugation (600 g, 10 min, room temp., with brake) and resuspended at a density of 1 × 10^8^ platelets/ml in HEPES-buffered saline (HBS; 135 mM NaCl, 10 mM HEPES, 3 mM KCl, 1 mM MgCl_2_, 0.34 mM NaH_2_PO_4_, pH 7.4, supplemented with 0.9 mg/ml d-glucose). Platelets were rested at 30 °C for 30 min prior to experimentation. For inhibition of COX, platelets were incubated with aspirin (300 μM) 30 min prior to being added to the Oxygraph chamber. Control samples were incubated with an equivalent amount of the vehicle (DMSO) for the same time. In contrast, oligomycin (or its vehicle, ethanol, as control) were added directly to platelets in the Oxygraph chamber, as indicated in the *Results* section.

### 2.3 Measurement of oxygen flux during platelet activation

Oxygen consumption in washed human platelets was measured using an Oxygraph-2k high-resolution respirometer (Oroboros Instrument, Innsbruck, Austria) in 2 ml glass chambers at a constant temperature of 37 °C and stirrer speed 750 rpm. Oxygen flux (*J*O_2_), which is directly proportional to oxygen consumption rate, was continuously recorded with a 2 s sampling rate using DatLab software 6.1 (Oroboros Instruments, Austria). Calibration at air saturation was carried out every day prior to experimentation, and all data were corrected for background instrumental oxygen flux in accordance to the manufacturer's instructions. Platelets (1 × 10^8^/ml in HBS with glucose, as described above) were added to the Oxygraph chambers and allowed to reach a stable baseline oxygen consumption rate prior to stimulation in the presence of CaCl_2_ (2 mM). All reagents injected into the chambers (CaCl_2_, thrombin, PAR agonists, oligomycin or vehicle control) were warmed to room temperature prior to addition.

### 2.4 Statistical analyses

Data are reported as mean ± standard error of the mean (SEM); the reported *n* value indicates the number of independent platelet preparations from different donors. Data were analysed by paired *t*-test for comparison of two conditions, and by RM two-way ANOVA with Sidak multiple comparison test for comparison of the effect of oligomycin in the absence or presence of aspirin.

## 3 Results

### 3.1 Thrombin triggers a rapid increase in oxygen flux in platelets

**Fig. 1:**
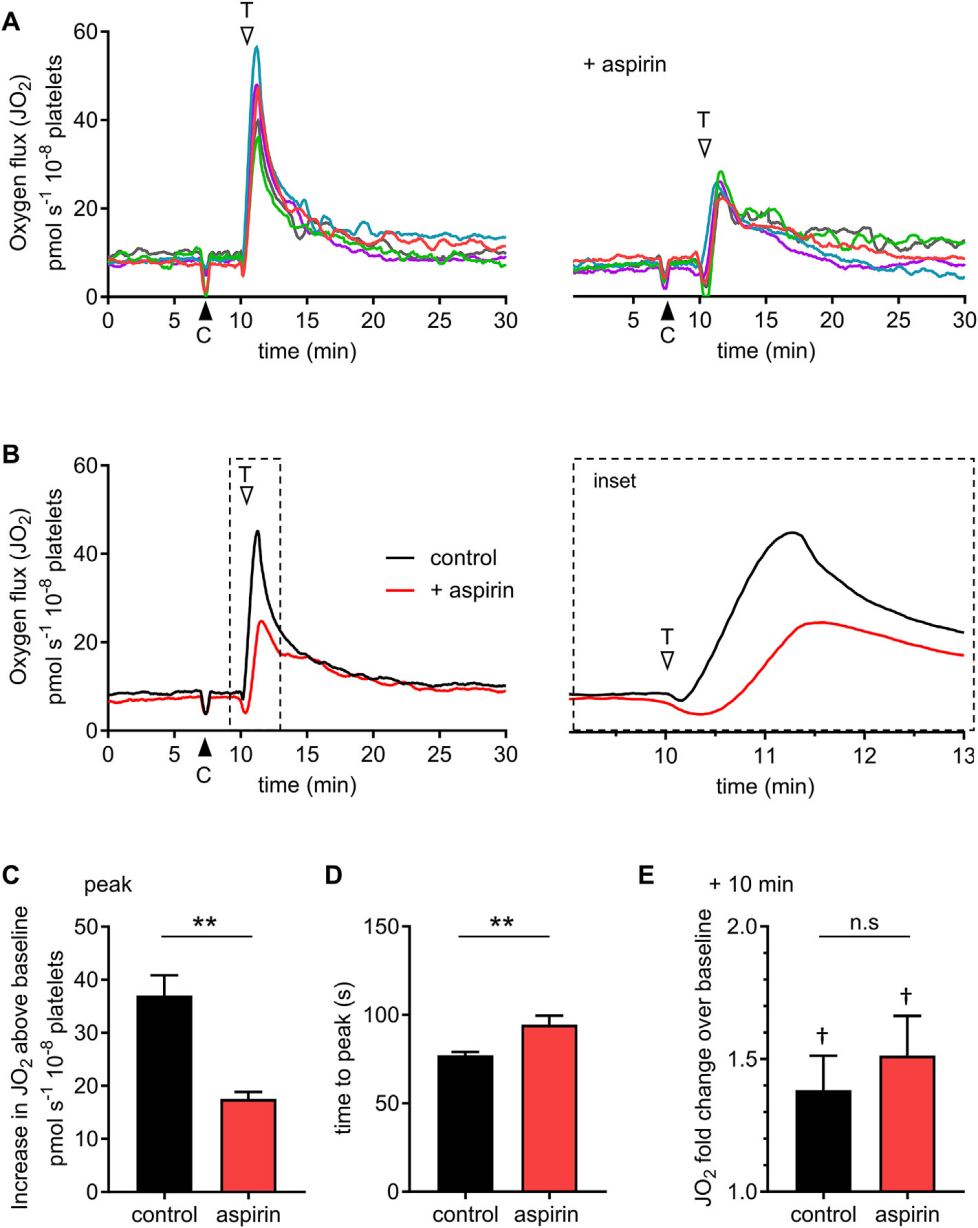
**Thrombin triggers a rapid increase in oxygen flux (*J*O**_**2**_**), which is partly inhibited by aspirin**. Washed platelets were treated with aspirin (300 μM) or its vehicle (DMSO, 0.1%; ‘control’) then stimulated with thrombin in an Oxygraph-2k high resolution respirometer. CaCl_2_ (final concentration, 2 mM) was added at the black arrowhead (‘C’). Thrombin (1 U/ml) was added at the white arrowhead (‘T’). In **A**, individual traces from platelet independent platelet preparations (different donors) are shown. The control (DMSO) is the left-hand panel, and aspirin-treated platelets are shown in the right-hand panel. Matched colours indicate the pair experiments. The mean signal (n = 5) is shown in **B**; the dashed region is expanded in the right-hand panel. The peak change in *J*O_2_ (above the baseline prior to thrombin addition) is shown in **C** and the time from thrombin addition to this peak is shown in **D** (mean + SEM; n = 5; **p < 0.01 for control vs. aspirin). The *J*O_2_ 10 min after thrombin addition is shown in **E**, reported as fold change over the baseline (n.s. not significant for control vs. aspirin; †p < 0.05 for fold-change at 10 min significantly different to pre-stimulation baseline). This measure was chosen to aid comparison with a previous report, as described in the *Results* section.

Washed platelets were treated with DMSO (0.1%; the vehicle control for aspirin – see next section) then stimulated with the physiological activator, thrombin (1 U/ml) in the presence of extracellular CaCl_2_ (2 mM). Extracellular oxygen concentration was monitored. This was converted to the oxygen flux, *J*O_2_, which is directly proportional to O_2_ consumption rate, and is shown in Fig. 1A–B. Prior to stimulation, *J*O_2_ was 8.5 ± 0.4 pmol s^−1^ 10^−8^ platelets. Thrombin triggered a rapid, transient increase in *J*O_2_. The peak increase in *J*O_2_ was 39.6 ± 5.6 pmol s^−1^ 10^−8^ platelets higher than the pre-stimulation baseline (Fig. 1C; a 5.4 ± 0.6 -fold increase) and occurred at 72.8 ± 2.9 s after stimulation (Fig. 1D). *J*O_2_ then decreased over the following 20 min. A previous study using the Seahorse XF [3] reported that thrombin stimulated an increase in oxygen consumption rate by ‘approximately 25%’ at the next reported point of measurement (8 min later). For comparison, in our experiments, after 10 min stimulation with thrombin, *J*O_2_ was 38.3 ± 13.0% higher than the pre-stimulation baseline (Fig. 1E; n = 5; p = 0.045).

### 3.2 COX activity contributes to the rapid increase in *J*O_2_

COX enzymes use oxygen in the generation of eicosanoids from arachidonic acid [24]. Arachidonic acid (500 μM) stimulated a rapid increase in *J*O_2_, which was inhibited by aspirin, a COX inhibitor (300 μM; Suppl. Fig. 1). To assess the contribution of COX to the thrombin-triggered increase in *J*O_2_, platelets were stimulated in the presence of aspirin (Fig. 1A, right hand panel). The peak increase in *J*O_2_ was significantly inhibited and delayed by aspirin (Fig. 1C–D). (The delay in reaching peak *J*O_2_ is clearly shown in the inset panel of Fig. 1B.) However, thrombin was still able to trigger an increase in *J*O_2_ that was 2.4 ± 0.3 -fold higher than the pre-stimulation baseline, indicating that thrombin also rapidly activates other sources of O_2_ consumption.

In contrast to the peak increase in *J*O_2_, the increased *J*O_2_ 10 min after stimulation was not significantly affected by aspirin (Fig. 1E; p = 0.93; n = 5).

### 3.3 Protease-activated receptor agonists rapidly increase JO_2_

**Fig. 2:**
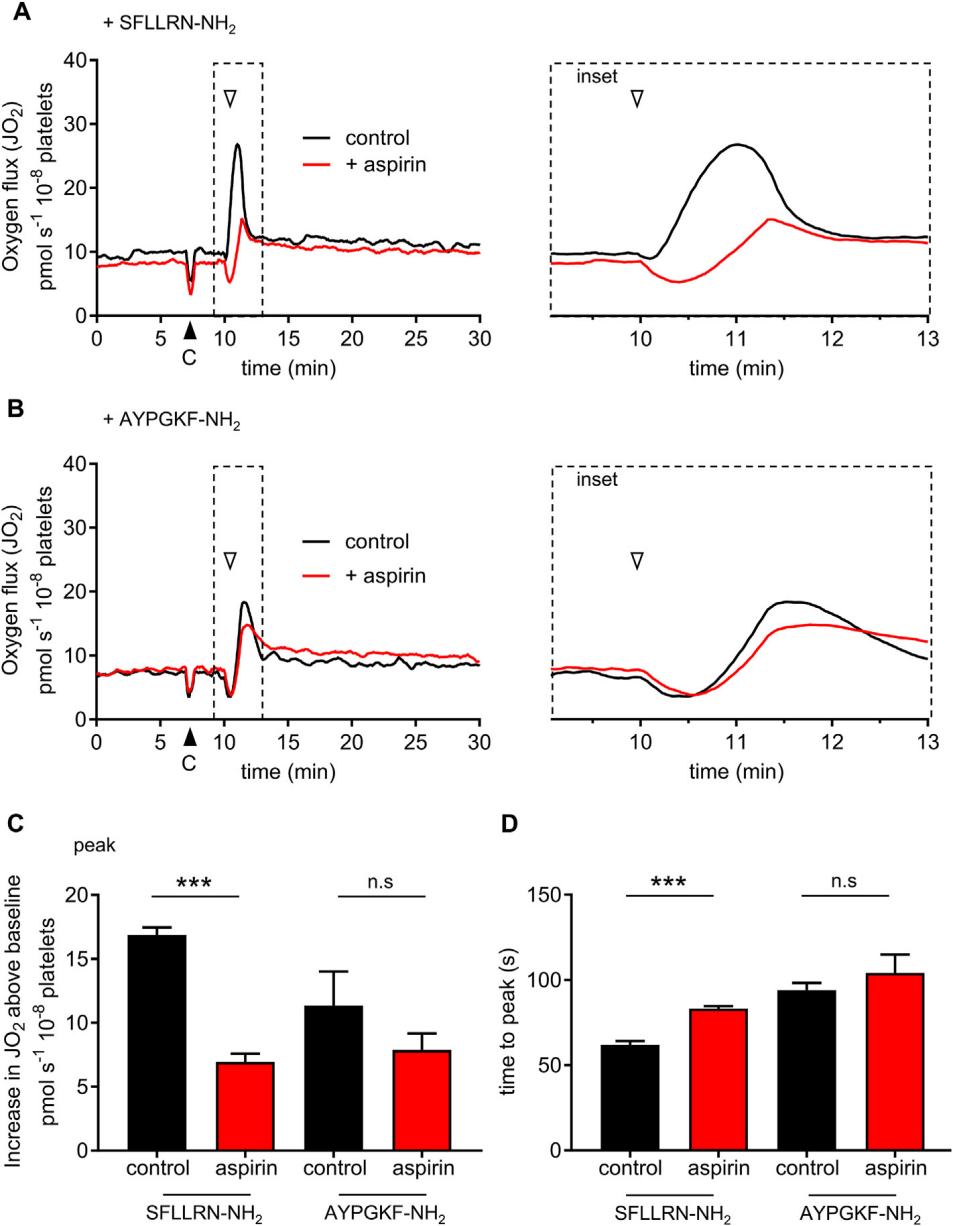
**Protease-activated receptor agonist peptides trigger a rapid increase in *J*O**_**2**_. Washed platelets, treated with aspirin or vehicle control as in Fig. 1, were stimulated with SFLLRN-NH_2_, a PAR1 agonist (A) or AYPGKF-NH_2_, a PAR4 agonist (B). The traces represent the mean signal (n = 5). The black arrowhead indicates addition of CaCl_2_. The white arrowhead indicates addition of agonist peptide. The dashed area is shown expanded as an inset panel to the right. The peak change in *J*O_2_ (above the baseline prior to thrombin addition) is shown in **C** and the time from agonist peptide addition to this peak is shown in **D** (mean + SEM; n = 5; ***p < 0.001 for control vs. aspirin, n.s. not significant; n = 5).

In human platelets, thrombin activates the protease-activated receptors, PAR1 and PAR4, and glycoprotein Ib (GPIb). It has been reported that thrombin-stimulated O_2_ consumption is dependent on GPIb, and that stimulation of PAR1 does not increase O_2_ consumption [25]. However, we observed a rapid and transient increase in *J*O_2_ when platelets were stimulated with SFLLRN-NH_2_ (10 μM) or AYPGKF-NH_2_ (200 μM), selective agonists of PAR1 and PAR4, respectively (Fig. 2A–B). Under these conditions, aspirin significantly inhibited the peak increase in *J*O_2_ in response to SFLLRN-NH_2_, from 16.9 ± 5.9 to 6.9 ± 0.7 pmol s^−1^ 10^−8^ platelets (n = 5; p < 0.001), but had a less consistent effect when platelets were stimulated with AYPGKF-NH_2_ (the peak increase in *J*O_2_ was 11.3 ± 2.7 pmol s^−1^ 10^−8^ platelets in the absence of aspirin, and 7.9 ± 1.3 pmol s^−1^ 10^−8^ platelets in aspirin-treated platelets; n = 5; p = 0.22). These data are summarised in Fig. 2B–D. 10 min after stimulation with SFLLRN-NH_2_, *J*O_2_ was 14.5 ± 3.9% above the pre-stimulation baseline (n = 5; p < 0.05), whereas 10 min after AYPGKF-NH_2_, *J*O_2_ was 16.0 ± 7.1% (n = 5; p = 0.85) above the pre-stimulation baseline.

### 3.4 The higher post-stimulation *J*O_2_ is inhibited by oligomycin

**Fig. 3:**
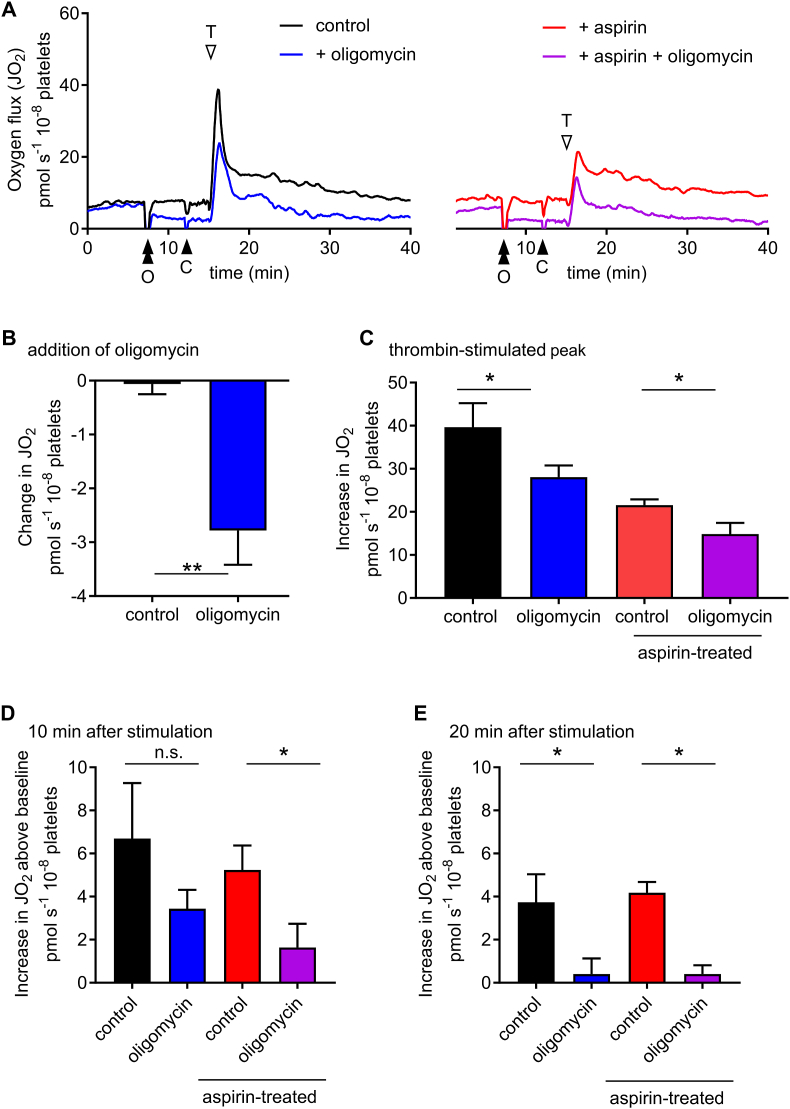
**Oligomycin inhibits *J*O**_**2**_**in unstimulated and stimulated platelets**. Washed platelets were treated with oligomycin (2.5 μM) or its vehicle (ethanol, 0.05%) as control at the point indicated by the double black arrowhead (‘O’). CaCl_2_ was then added (black arrowhead, ‘C’) followed by thrombin (white arrowhead, ‘T’). The traces show mean signal (n = 5) from platelets with either DMSO (0.1%; left-hand panel) or aspirin (300 μM; right-hand panel). The immediately decrease in *J*O_2_ following addition of oligomycin is shown in **C** (mean + SEM; n = 5; **p < 0.01). The mean peak increase in *J*O_2_ above baseline prior to thrombin addition (but after oligomycin or its control) is shown in D (mean + SEM; n = 5; *p < 0.05 for control vs. ethanol). The increases in *J*O_2_ 10 or 20 min after thrombin addition are shown in **D** or **E**, respectively.

To determine the contribution of OXPHOS to the thrombin-stimulated changes in *J*O_2_, we treated platelets with oligomycin (2.5 μM), an inhibitor of the mitochondrial F_1_F_o_-ATP synthase. Oligomycin rapidly decreased baseline *J*O_2_ (Fig. 3A–B; p = 0.011 compared with vehicle control). The peak increase in *J*O_2_ was also partially inhibited by oligomycin, either in the absence or presence of aspirin (Fig. 3A and C). The increase in *J*O_2_ 10 min after stimulation was inhibited by oligomycin in the presence of aspirin (Fig. 3D). By 20 min post-stimulation, aspirin had no effect and oligomycin completely inhibited the remaining increase in *J*O_2_ (note that this is relative to an already-inhibited baseline). This indicates that oligomycin-sensitive OXPHOS contributes to the rapid, transient increase in *J*O_2_, and to the sustained smaller increase. Notably, other sources of O_2_ consumption also contribute to the peak change in *J*O_2_, as the rapid increase in oxygen consumption was not completely inhibited by aspirin and oligomycin in combination.

## 4 Discussion

Platelet stimulation with thrombin triggers a rapid, transient increase in *J*O_2_. The peak of this increase occurred slightly later than 1 min after stimulation and then declined to a new baseline over the following 10–20 min. The rapid increase in *J*O_2_ occurs on the same timescale as more-commonly measured platelet functions, such as aggregation or granule secretion and shows striking similarities to thrombin-triggered intracellular Ca^2+^ signalling [e.g. Ref. [26]]. In contrast, published reports of platelet O_2_ consumption using the Seahorse XF have so far missed this rapid increase in *J*O_2_ [3]. Extracellular flux analysis has shown a relatively small increase in oxygen consumption rate (OCR) of around 25%. This is similar in magnitude to the approximately 38% average increase in *J*O_2_ that we observed 10 min after stimulation, after most of the peak increase in *J*O_2_ had subsided. This suggests that although the published methods using Extracellular Flux Analysis may be useful for investigating more sustained changes in oxygen consumption in platelets following stimulation, high resolution respirometry is more useful for investigating the rapid, transient burst of O_2_ consumption that occurs immediately following platelet stimulation.

In this study, the platelets were isolated from plasma and resuspended in HEPES-buffered saline with 5 mM glucose as the exogenous source of metabolic substrates. This is a relatively common approach for many platelet signalling studies [27], and an advantage of this approach is that data can be readily compared with published data on platelet aggregation, granule secretion and intracellular signalling. However, the effect of adding other exogenous substrates, such as glutamine, pyruvate or fatty acids, could be investigated in this protocol, and may affect the extent of increase in OXPHOS following stimulation [3].

The rapid increase in *J*O_2_ in response to thrombin was also observed when platelets were stimulated with peptide agonists of PAR1 or PAR4. This is not very surprising, since thrombin triggers intracellular signalling in platelets by activating these receptors [28]. The increase in *J*O_2_ 10 min after stimulation was statistically significant for PAR1 stimulation, though this was relatively small, and may not be robustly observed under different experimental conditions. This may explain why a previous report found no increase in OXPHOS in response to PAR1 stimulation [25].

The pioneering studies of the 1970s onwards attributed the rapid increase in O_2_ consumption rate to COX activity [6,7]. Although we broadly replicated these observations in this study, our high-resolution respirometry shows a more complex picture. In our study, aspirin significantly inhibited the rapid, peak increase in *J*O_2_, consistent with the previous reports. However, with high resolution respirometry, we observe that there is still a substantial increase in *J*O_2_, indicating that there are other mechanisms of O_2_ consumption occurring. This demonstrates the benefit of high-resolution respirometry over Clark-type electrodes in understanding the changes in O_2_ consumption during platelet activation. Similarly, in the early studies, oligomycin-sensitive OXPHOS accounted for the basal O_2_ consumption in unstimulated platelets but did not significantly contribute to the increased O_2_ consumption in thrombin-stimulated platelets. We also demonstrate that the baseline O_2_ consumption could be inhibited by oligomycin and likely represents the contribution of OXPHOS to ATP synthesis in unstimulated platelets. However, we also show that oligomycin inhibits the rapid peak increase in *J*O_2_, independently of COX activity. This might represent a rapid, transient activation of OXPHOS, which might support ATP-dependent events during the earliest stages of platelet activation. It could also represent another pathway of O_2_ consumption that is reduced by the inhibition of baseline ATP synthesis. The remaining O_2_ consumption, which is not inhibited by aspirin or oligomycin, could involve other O_2_ consuming pathways that are known to be activated in platelets, such as 12-lipoxygenase, xanthine oxidase or NADPH oxidase [[29], [30], [31], [32]].

In summary, we show that high-resolution respirometry can be used to investigate the kinetics of changes in O_2_ consumption rate in stimulated platelets, across a timescale that is relevant to the rapid activation of platelets. Our data show that OXPHOS is a major contributor to O_2_ consumption in unstimulated platelets. Following stimulation, O_2_ is rapidly consumed in a COX-dependent manner, as previously reported. However, the high resolution of this approach shows that other pathways of O_2_ consumption are also rapidly activated. This gives a more complex picture of the rapid kinetics of changes in O_2_ consumption than was possible with Clark electrodes or has been reported with Extracellular Flux Analysis. High resolution respirometry is therefore a useful tool to investigate the rapid kinetics of changes in O_2_ consumption during platelet activation.
