# Supplementary material for: Rapid kinetics of changes in oxygen consumption rate in thrombin-stimulated platelets measured by high-resolution respirometry
Source: Biochem Biophys Res Commun. 2018 Sep 18;503(4):2721–7. doi: 10.1016/j.bbrc.2018.08.031 (PMC6142173; doi:10.1016/j.bbrc.2018.08.031)
Supplement: Supplementary Fig. 1 [file mmc1.docx]

Supplementary Figure 1

C

AA

**Supplementary Figure 1:**

Washed platelets were treated with aspirin (300 µM) or its vehicle (DMSO, 0.1%; ‘control’) then stimulated with arachidonic acid (500 µM). CaCl_2_ (final concentration, 2 mM) was added at the black arrowhead (‘C’). Arachidonic acid was added at the white arrowhead (‘AA’). The traces show the mean signal (n = 3).
